# 3D Simulation of an Audible Ultrasonic Electrolarynx Using Difference Waves

**DOI:** 10.1371/journal.pone.0113339

**Published:** 2014-11-17

**Authors:** Patrick Mills, Jason Zara

**Affiliations:** School of Engineering & Applied Science, The George Washington University, Washington, DC, United States of America; University of Zurich, Switzerland

## Abstract

A total laryngectomy removes the vocal folds which are fundamental in forming voiced sounds that make speech possible. Although implanted prosthetics are commonly used in developed countries, simple handheld vibrating electrolarynxes are still common worldwide. These devices are easy to use but suffer from many drawbacks including dedication of a hand, mechanical sounding voice, and sound leakage. To address some of these drawbacks, we introduce a novel electrolarynx that uses vibro-acoustic interference of dual ultrasonic waves to generate an audible fundamental frequency. A 3D simulation of the principles of the device is presented in this paper.

## Introduction

The loss of speech is a huge loss of self; who we are is intimately connected to our “voice.” Every year, many thousands of people worldwide lose the ability to speak after undergoing a laryngectomy, typically for treatment of cancer. In developed countries, organ preservation has significantly reduced the number of total laryngectomies performed; in developing countries and for late-stage cancer, a total laryngectomy is still the preferred standard of care [1]. In a total laryngectomy, the entire larynx is removed including the vocal folds – which are made up of mucosal layers and the vocalis muscle – and the cartilage structures which support them [2]. Fortunately, in most cases, patients still have functioning vocal tracts and vocalization should be possible as long as a suitable sound source is provided [3]. While prosthetic surgical voice restoration (PSVR) is reducing the number of individuals who depend on external devices, at some point in their recovery, most patients will use an electrolarynx (EL) for some period of time [4].

For healthy speakers, voicing sounds are produced by the vibration of the vocal folds induced by glottal air flow. In the absence of air flow, the vocal tract pressure is at equilibrium and the glottis is closed; as air is forced out, the pressure within the vocal tract increases and the glottis opens. During speech, the laryngeal muscles tighten the vocal folds creating a lower pressure region which causes the glottis to close. The repeated opening and closing generates a pressure wave at the fundamental frequency, F_0_; the discontinuity caused by the closure gives rise to harmonics of F_0_ [5]. Formby and Monsen [6] point out that these harmonics provide peaks in the speech spectra that are important for speech intelligibility. Markel and Gray [7] state that a first order estimate of the harmonic roll off rate can be modeled by −12 dB/octave. Stemple *et al.* [8] analyzed 240 healthy speakers making a sustained neutral/a/vowel and reported that females have a mean F_0_ of 192 Hz with a range of 137–634 Hz, and men have a mean of 106 Hz with a range of 77–482 Hz; children have a higher F_0_ with boys almost double adult males.

Common external ELs in use today consist of a cylinder containing an electromagnetic actuator with an attached piston, driving electronics, a battery, user controls and a floating coupling disc. The user holds the device firmly against the neck, and when activated, the piston repeatedly strikes the coupling disc delivering pressure waves into the soft tissue. The pressure waves mechanically couple with the vocal tract and generate the F_0_ necessary for creating vowels, without which speech is not possible [9]. These devices suffer from poor frequency control due to the nonlinear character of their impulse driver [10]. The limited F_0_ selection is challenging for females who often remark that EL speech makes them sound more male [11]. Furthermore, the EL pressure wave has a deficit of low-frequency components [12]. Acoustic sound leakage, commonly known as self-noise, results in a steady background noise that is distracting to listeners and makes using voice communications systems difficult [13]. Mallis *et al.* [14] found that after a total laryngectomy, 23.9% of patients in their study felt embarrassed about their alaryngeal voice, decreased their social interactions, and experienced negative effects on their sex lives. 56.5% had trouble communicating with strangers and 78.3% had difficulties on the telephone. An overwhelming number reported negative effects on their work life.

**Figure 1:**
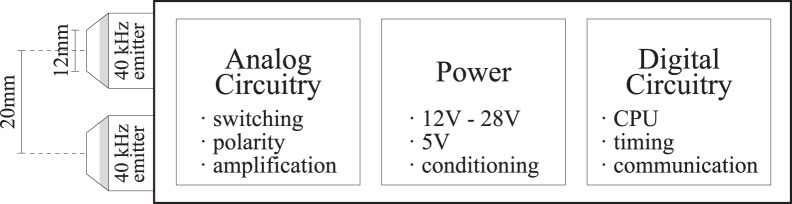
Proposed device schematic.

We have designed a new type of EL that uses interfering ultrasonic waves to generate a F_0_. A schematic of a basic prototype is shown in Figure 1. We hypothesize that our device will offer many advantages over the classic piston driven devices including a wide range of fundamental frequencies, excellent frequency control, and low acoustic leakage. Like a classic EL, the device is pressed against the neck and an activation button pressed. Each transducer then emits a slightly different frequency which penetrates the tissue and interferes. From basic wave theory, we know that we can control the location of the maxima by varying the separation, angle and phase of the two emitters. By using two separate waves, we minimize the distortion of the final output wave as it will be the amplitude modulation of the two input waves.

In this paper, we present a linearized three-dimensional Finite-Difference Time-Domain (FDTD) simulation of our novel EL implementation that uses two point sources to generate a F_0_ and integral harmonics in the vocal tract required for speech restoration. For the model, we use the AustinMan v1.1 Partial Body male model [15] voxels (volume pixels) at a resolution of 1 mm^3^. This model includes slices from the top of the head down to the upper chest and consists of over 101 million individual voxels. Each voxel is assigned a tissue type to which we have assigned individual properties for density, speed of sound, and attenuation. The result of the simulation is a sound wave measured within the vocal tract. We compare the results of our simulated device with a series of synthetic glottal pulses and measurements of a popular EL and show that our device closely mimics a natural excitation.

## Theory

The goal of our simulation is to model two ultralow-frequency (25 kHz–100 kHz) ultrasound beams at slightly different frequencies as they enter the body, pass through soft tissue, and interfere. When wave interference causes the sound pressure level (SPL) to vary at a given location at a stable frequency, this new frequency is called the beat frequency and is defined as the difference between the two input frequencies [16].

In order to show that a beat frequency can be generated at a location and frequency useful for speech formation, we need to simulate the ultrasound sources, the waves, the tissue and the airway with as few assumptions as possible. We use the FDTD method as it is able to calculate the wave over complex geometry. Refraction, reflection and diffraction are included in the propagation calculations as no physical approximations are made.

Numerical simulation of acoustic fields using the FDTD method in the time domain relies on the linearized equations of motion and continuity: [17]

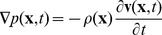
(1)

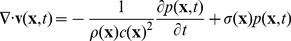
(2)where **v** is the field vector of particle velocity, *p* is the acoustic pressure, ρ is the spatially dependent mass density, *c* is spatially dependent speed of sound, and σ represents the total losses of the acoustic energy due to absorption by thermal conduction and fluid viscosity.

The linearized equations model longitudinal waves; modeling of mode conversion to shear waves at boundaries is bundled into the loss coefficient. Luo *et al.* [18] describe attenuation as a result of scattering and absorption where absorption is due to shearing motions, conduction-based heat loss, and chemical relaxation processes. They state that sound absorption is a relaxation process that occurs when the equilibrium constant of a chemical reaction is affected by temperature and/or pressure changes. They report that the majority of low-frequency ultrasound absorption in tissue can be accounted for by chemical relaxation and that absorption is proportional to the square of the frequency. Sehgal and Greenleaf [19] state that the relaxation time for tissue is sub-microsecond, and for ultralow-frequency ultrasound the effects are negligible.

In order to discretize the wave calculation, we implemented a standard Yee FDTD (2,4) central difference algorithm which is second-order in time and fourth-order in space [20]. For an in-depth review of FDTD, see Schneider [21]. Since pressure is a scalar field, time zero consists of the initial pressures and the simulation starts with calculating the velocity field. At each time step, the calculation alternates between updating velocity and pressure.

Numerical precision is an important consideration in any discretized calculation. In order to avoid spatial and/or spectral aliasing, the Nyquist criterion, which requires the time step to encompass at least two points per frequency period, must be satisfied. Also, the spatial spacing should be 5–10 times less than the smallest signal wavelength. The maximum stable time step can be calculated from the Courant number [22].

One complication with the FDTD method is that as wave propagation reaches the domain edge, phantom reflections are generated. To reduce these anomalies, it is necessary to highly attenuate signals as they approach the simulation boundary. There are many methods for implementing these absorbing boundary conditions; we used Mur’s “Absorbing Boundaries”, [23] as most of the energy in our model remains within the soft tissue and the parallel implementation is much simpler.

## Methods

### The Model

**Figure 2:**
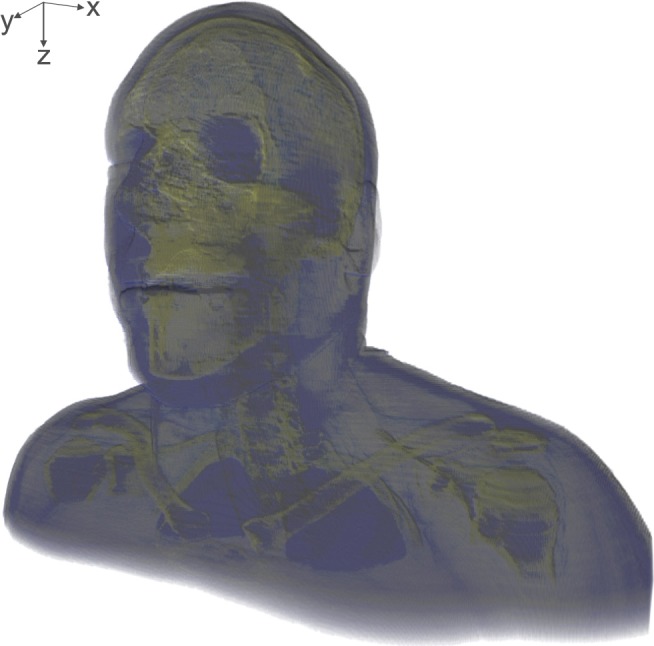
Rendering of the AustinMan voxels used in the FDTD simulation.

The 3D upper body model used for our simulation is shown in Figure 2 using a right-handed coordinate system. The model consists of segmented and labeled voxels extracted from high-resolution cryosection images from the National Library of Medicine’s Visible Human Project. Our model consists of the first 320 vertical slices, each one cropped to a 512×288 grid. As each voxel represents a volume of 1 mm^3^, we have a simulation area of 512 mm wide×288 mm deep×320 mm high. The volume surrounding the model is treated as air; the other 13.5 million voxels consist of 26 tissue types. Table 1 shows each tissue type, the number of voxels of this type present in the model, and the material properties used in the simulation and where they were found in the literature.

**Table 1:** Tissue Properties.

ID	VoxelCount	Name^1^	Density(kg/m^3^)	Speed ofSound (m/s)	AttenuationdB/cm/MHz	Reference^2^
0	87,692,282	Air @ 25°C,60% Rel. Hum	1.2	347	0.012	[24]; [24]; [24]
1	29,979	Int.Air @ 30°C,90% RH [25]	1.15	351	0.012	[24]; [24]; [24]
12	822,305	Brain(Grey Matter)	1045	1562	0.625	[26]; [27]; [27]
28	159,667	Blood Vessel	1102	1584	0.2	[26]; [24]; [28]
32	492,584	Brain(White Matter)	1041	1562	1.05	[26]; [27]; [27]
72	324	Cornea	1051	1588	0.5	[26]; lin int [28]; *typ*
80	109,897	Cerebrospinalfluid	1007	1528	0.5	[26]; lin int [28]; *typ*
84	725,361	Bone (Cortical)	1908	2740	20	[26]; avg [24]; [28]
92	619,919	Bone (Marrow)	1029	1560	5	[26]; lin int [24]; [28]
96	2,180	Eye (Sclera)	1032	1560	0.5	[26]; lin int [28]; *typ*
104	474,305	Lung	722	1125	0.5	avg [26]; lin int [28]; *typ*
112	78,158	Cartilage	1100	1729	0.5	[26]; lin int [28]; *typ*
116	508	Eye (lens)	1076	1656	2.0	[26]; avg [24]; [29]
124	6,322	Dura	1174	1563	0.5	[26]; lin int [28]; *typ*
136	1,706,776	Fat	911	1450	0.65	[26]; [28]; [28]
144	12,489	Esophagus	1040	1562	0.5	[26]; lin int [28]; *typ*
152	176,288	Gland	1050	1570	0.5	[26]; lin int [28]; *typ*
156	51,743	Tongue	1090	1562	0.5	[26]; lin int [28]; *typ*
168	7,642	Lymph	1035	1570	0.5	[26]; lin int [28]; *typ*
172	14,049	Teeth	2063	4695	20	[26]; avg [24]; [27]
180	5,492,061	Muscle	1090	1579	3.3	[26]; avg [24]; [29]
184	47,956	Spinal cord	1075	1562	0.5	[26]; lin int [28]; *typ*
192	19,903	Nerve	1075	1562	1.55	[26]; lin int [28]; avg [27]
196	13,281	Eye(vitreous humor)	1009	1528	0.1	[24]; avg [24]; [29]
204	911,595	Skin	1100	1729	0.8	[24]; [24]; [28]
208	10,926	Mucosa	1102	1570	0.5	[26]; lin int [28]; *typ*
216	11,325	Trachea	1080	1729	0.5	[26]; lin int [28]; *typ*
220	1,347,600	Tendon/Ligament	1142	1729	4.7	[26]; lin int [27]; [27]
		Soft tissue	1000	1540	0.5	NIST; [30]; *typ*

1 Rel. Hum = RH = Relative Humidity; Int Air = Air in Internal Cavity.

2 *lin int* = linear interpolation; *typ* = typical; *avg* = average.

We have not made any modifications to the AustinMan v1.1 Partial Body male model data set in order to allow other investigators to validate, compare and extend our results. While it is possible to model a laryngectomy by removing the larynx, cartilage and surrounding tissue, we consider simulations through this tissue a worst case scenario. Similarly, radiation therapy usually results in fibrosis and edema which hardens the neck tissue; these effects have been ignored in this simulation as the effects often subside and the neck tissue softens or a suitable alternative location for device placement can be found [13]. Given that the larynx in our model is intact, we have shifted the transverse location of our inputs down into the subglottal region; as our goal is to determine whether or not a fundamental frequency can be generated in the vocal tract using a beat frequency, we do not view this as a problem.

### The Simulator

The simulator is written in C++ and Compute Unified Device Architecture (CUDA) and based on a room acoustic simulator by Ola Vikholt [31]. Given that we need to evaluate pressure and velocity as double-precision floating point values at over 47 million nodes for each time step, it is important that the CUDA code be as optimal as possible. Therefore, the code was enhanced to accommodate the AustinMan model by extending the material handler from a fixed three material system to a user defined number with file based material properties and voxel import, optimize the kernels to minimize synchronization and maximize work per call, minimize kernel memory management overhead, minimize memory transfer between the GPU and CPU, and maximize GPU occupancy for parallel execution.

The simulations were run on a Windows 7 64-bit machine with an Intel i7 2.8 GHz 8 core processor, 24 GB of RAM, and a NVIDIA GeForce GTX 660 Ti video card with driver version 5.0 and runtime version 4.2. The video card is a CUDA Capability 3.0 card with 3 GB of global memory, 1344 cores running at 1.1 GHz, a warp size of 32, and a maximum of 1024 threads per block.

### Simulator Stability

In order to determine stable operating parameters for the simulation, we first define the source frequencies. As our ultimate goal is to implement a portable, cost-effective physical device with as high a SPL difference wave as possible, we use frequencies in the 40 kHz range. These frequencies are high enough to be above human and domesticated animal hearing ranges, low enough to have minimal tissue absorption, and are generally considered safe below 110 dB [32].

Given the fixed resolution of the AustinMan model, our spatial step size, δ, is 1 mm in all three dimensions. To ensure valid results, δ must be 5–10 times less than the smallest wavelength. Referring to Table 1, air has the lowest speed of sound; combined with our highest frequency, we have a minimum signal wavelength of 8.6 mm which is within the stable zone. From Table 1, the teeth have the highest speed of sound; therefore, the maximum stable time step as determined by the Courant number is 122.97 ns. As we will be recording output at 96 kHz, we lower the time step to 122.5 ns, which gives us a sampling frequency of 8.16 MHz or 85 times our output sampling rate. We could achieve over a 3x speedup if we simply used soft tissue parameters everywhere; however, we have opted for the more computationally-intensive but potentially more accurate option of full tissue properties.

### Transducers and Microphones

**Figure 3:**
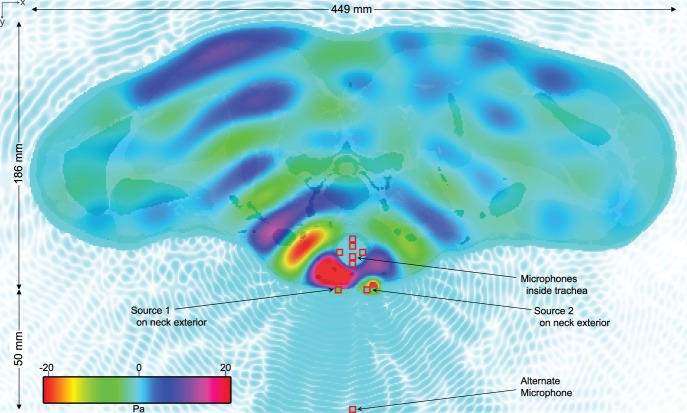
Transverse view of simulation pressures at z = 278 mm, t = 63.7 ms at 200 Hz.

Commercially available low-frequency ultrasonic transducers have a typical radius of 5–10 mm. We model 6 mm radius ultrasonic transducers as sinusoidal hard point sources; to simulate the hemispherical nature of the transducers we included a 4 mm polypropylene foam baffle behind the sources. Figure 3 shows the two sources and seven microphones as square icons. Source 1 is located at (249 mm, 202 mm, 278 mm) and source 2 at (269 mm, 202 mm, 278 mm).

Based on the findings of Stemple *et al*. [8], we have modelled three sets of fundamental frequencies: 100 Hz, 200 Hz, and 400 Hz. These frequencies cover normal speech in the vast majority of men, women and children. Based on the mathematics involved, we anticipate only minor pressure differences, ±5 dB SPL, as the beat frequency changes. The technique should work equally well over a wide range of beat frequencies. In all simulations, source 1 is 40 kHz @ 0° phase and source 2 is 90° phase; both sources have amplitudes of 105 dB. For 100 Hz, source 2 is 39.9 kHz; for 200 Hz, it is 39.8 kHz; and for 400 Hz, it is 39.6 kHz.

We modelled six recording microphones within the trachea located at: (259 mm, 167 mm, 278 mm), (259 mm, 172 mm, 278 mm), (250 mm, 176 mm, 278 mm), (266 mm, 184 mm, 278 mm), (259 mm, 179 mm, 278 mm), (259 mm, 185 mm, 278 mm). To examine the acoustic leakage, we also placed a microphone located at (259 mm, 252 mm, 278 mm) which is 5 cm from the device/neck interface.

### Glottal Waveform

There are a number of well-known glottal pulse models including: Rosenberg, Fant, Liljencrants-Fant, and Klatt [33]. Each model uses different parameters to represent the glottal cycle and therefore results in slightly different waveforms. For our purposes, we are interested in the pulse period, and the opening and closing phases; in particular, we want a continuous function with a discontinuous first-derivative as this combination results in the asymptotic decay of 12 dB/octave found in natural speech [34]. One of the simplest models with these properties is the Rosenberg pulse shape type C [35]. We used an amplitude of 105 dB, the opening time T_P_ = 40%, and the closing time T_N_ = 16% for each of the three fundamental frequencies.

We are interested in the excitation pulse only, not the vocal tract resonances/formants as these are physiological and for a given position/configuration will be the same irrespective of the excitation [36].

### Classic Electrolarynx

For comparison to a typical classic piston electrolarynx, we followed Qi and Weinberg [12] and utilized the popular Servox Inton. We recorded the device at 96 kHz in Adobe Audition using an Edirol UA-25 digitizer. Sound level readings were made with a Sper Scientific 850014 sound meter in dBC slow mode [37].

The device has a limited range of frequencies, approximately 70–260 Hz, so we were not able to generate data for comparison with the 400 Hz simulation.

## Results

The simulation for each beat frequency was run for over 520 K iterations or 63.7 ms of simulated time. This is more than enough time for the pressure waves to propagate throughout the entire model and reach dynamic equilibrium and to capture multiple cycles of F_0_. At the end of each simulation, the double-precision floating point pressure measurements at the microphone locations were exported to separate 96 kHz 16-bit PCM wave files. The shading in Figure 3 indicates the intensity of the pressure at each location in the transverse slice at z = 288 mm at the end of the simulation for the 200 Hz beat frequency.

**Figure 4:**
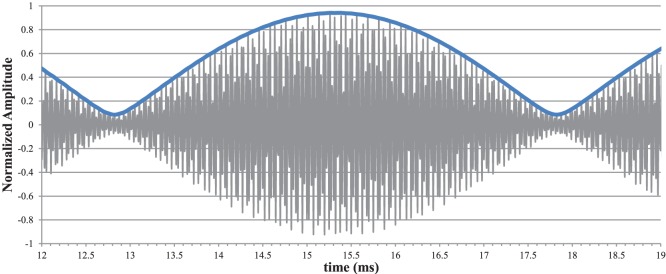
Recorded Waveform. The recorded waveform showing the amplitude modulation indicative of a beat frequency and an overlay of the demodulated signal showing the 200 Hz beat frequency.

Viewing the time-domain amplitude waveform, the beat frequency presents as an amplitude modulation of the two input frequencies. In order to demodulate the low-frequency signal for spectral analysis, we take the envelope of the waveform using a Short-time Fourier Transform (STFT) version of the discrete Hilbert Transform [38]. Examples of the recorded and demodulated signals are shown in Figure 4.

**Figure 5:**
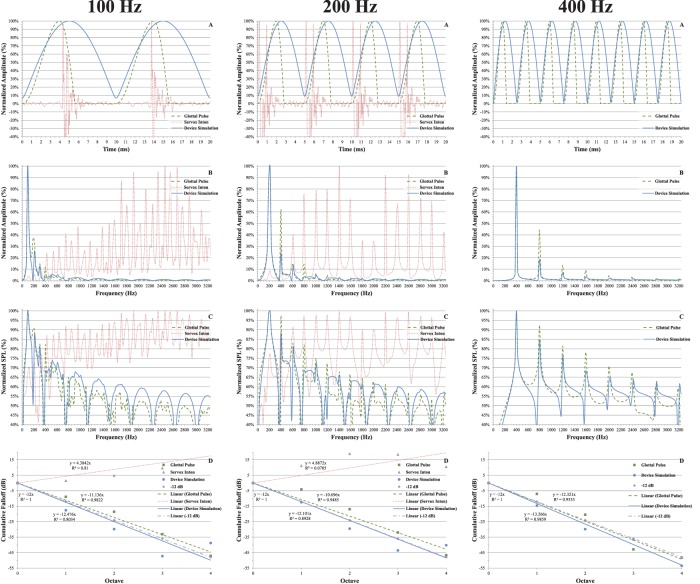
Excitation Pulse Comparisons. Comparison of peak normalized synthetic glottal pulses based on Rosenberg model (dashed green line), beat frequency from simulated device (solid blue line), and a Servox Inton piston electrolarynx (dotted red line). **A**. time domain excitation waveform. **B**. spectrum, **C**. SPL spectrum *(ref. 20* *µPa)*, **D**. Roll off per octave.

In Figure 5, we have arranged a series of four graphs (A, B, C, D) comparing the synthetic glottal pulse train (dashed green line), the simulated device (solid blue line), and the electrolarynx (dotted red line) for each F_0_. For each graph, the inputs have been normalized to equal peak amplitudes.

The graphs in 5A show the excitation pulse in the time domain. If the pulse generated by the device simulation was an actual glottal pulse, it would be considered breathy as there would be little glottal closure; however, as the excitation will be imparted mechanically, there will be no breathy effect.

The graphs in 5B show the spectrum of each excitation pulse. As noted by Qi and Weinberg [12], the electrolarynx suffers from a deficit of low-frequency energy which can be seen by the substantial dropoff in magnitude near the fundamental frequency. The graphs in 5C show the spectrum as a plot of SPL with a reference of 20 µPa. Both 5B and 5C show that the spectrum of the electrolarynx does not resemble the glottal pulse, while the device simulation is a very close approximation. To quantize the comparison, we ran a correlation between pairs of the normalized spectral magnitudes from 5B; Table 2 shows the results which support the conclusion that the simulated device generates a spectrum closer to a glottal pulse than does a classic electrolarynx.

**Table 2:** Correlation between normalized spectrum magnitudes.

FundamentalFrequency	Simulated GlottalPulse vs. Electrolarynx	Simulated Glottal Pulsevs. Device Simulation
100 Hz	−0.16	0.94
200 Hz	0.06	0.96
400 Hz	N/A	0.97

The graphs in 5D show the harmonic roll off. As stated earlier, natural speech has a roll off around −12 dB/octave. The electrolarynx increases with frequency; in fact, Weiss *et al.* [39] reported that a roll off of −14±2 dB/octave occurs as the frequency decreases, completely opposite of a natural spectrum. Table 3 shows the mean and standard deviation for the harmonic roll off.

**Table 3:** Harmonic roll off.

FundamentalFrequency	Simulated GlottalPulse (dBC)	DeviceSimulation (dBC)	Electrolarynx (dBC)
100 Hz	−11.8±2.5	−9.7±10.7	5.7±4.5
200 Hz	−11.7±4.4	−10.1±7.9	2.6±7.4
400 Hz	−12.1±6.7	−13.4±4.0	N/A

Table 4 shows the comparison of self-noise between the EL and the device simulation. Lower acoustic leakage will increase listener comprehension. Furthermore, as the primary waveforms are above human hearing, most communications devices will filter such noise as part of their standard digitization process.

**Table 4:** Self-noise output magnitudes for alternative microphone location in Figure 3.

Fundamental Frequency	Device Simulation (dBC)	Electrolarynx (dBC)
100 Hz	63.9±2.0	87.6±0.1
200 Hz	64.0±1.9	85.7±0.1
400 Hz	63.0±1.9	N/A

## Discussion

Our simulation shows that a beat frequency can indeed be generated within soft tissue and that, given appropriate inputs, this frequency can be made to occur within the vocal tract at a frequency appropriate for speech formation. Table 5 shows the magnitude of the outputs from the simulation. The magnitude was calculated by averaging the six microphone outputs and computing the standard error; this provides a good estimate of the pressure wave within the trachea as it varies due to location as in a canonical tube but also due to the natural deviations in the tissues.

**Table 5:** Device simulation output magnitudes for locations in Figure 3.

Fundamental Frequency	Device Simulation (dBC)
100 Hz	63.9±2.0
200 Hz	64.0±1.9
400 Hz	63.0±1.9

Coleman *et al.* [40] report that the minimum SPL at 15.25 cm for F_0_ is 48 dB and the maximum controlled SPL observed was 126 dB. Schindler *et al.* [41] observed tracheoesophageal voicing at 30 cm as having a minimum of 50±4.8 dB and a maximum of 68±4.7 dB. The vocal tract is a filter which spectrally shapes the fundamental frequency into the voice, amplifying frequencies near resonances and dampening others [42], [43]. However, even if we assume the worst case and apply the inverse square law where the energy dissipates in all directions proportionally to the distance [44], the observed levels are still within the error bounds of both studies.

Two of the major complaints with classic piston-driven ELs are the difficulty and range of frequency control and the self-noise generated by the devices. Given the method used to produce the F_0_ in our simulation, we anticipate that nearly linear control over the normal range of fundamental frequencies of men, women, and children should be possible. Even with low-grade ultrasonic transducers a range of 1 kHz should be possible, which exceeds any documented vocal fundamental frequency we have seen. For women in particular, our device’s ability to generate even very high fundamental frequencies would allow for a more natural feminine sounding voice.

Our simulation’s method for generating audible sound results in much lower self-noise. Only a small portion of the output will interfere outside the body and result in audible noise; the rest is outside the range of human hearing. Furthermore, unlike a classic EL where effective shielding is difficult; shielding in an ultrasonic device should result in a significant reduction in radiated noise. Further testing is necessary to test this hypothesis and determine to what extent it may be effective.

The results of our 3D FDTD simulation offer encouraging evidence that an EL based on ultrasonic difference waves will work and offer performance that has been difficult to achieve in other implementations. We have modeled the upper body as accurately as possible, and have found no factors in our simulations that would indicate an impediment to physical implementation. The comparison between the synthesized glottal pulse, the device simulation and a classic EL show how much more closely our device mimics a natural voicing excitation than does the EL.
